# Predefined Time Synchronization of Multi-Agent Systems: A Passivity Based Analysis

**DOI:** 10.3390/s23083865

**Published:** 2023-04-10

**Authors:** Vinay Pandey, Eram Taslima, Bhawana Singh, Shyam Kamal, Thach Ngoc Dinh

**Affiliations:** 1 Department of Electrical Engineering, Indian Institute of Technology (BHU), Varanasi 221005, India; 2 School of Electronics, Electrical Engineering and Computer Science, Queen’s University Belfast, Belfast BT9 6SB, UK; 3 Conservatoire National des Arts et Métiers (CNAM), CEDRIC-Laetitia, 292 rue Saint Martin, CEDEX 03, 75141 Paris, France

**Keywords:** finite-time stability, passivity, multi-agent systems, predefined-time stability

## Abstract

This paper deals with the predefined-time synchronization for a class of nonlinear multi-agent systems. The notion of passivity is exploited to design the controller for predefined-time synchronization of a nonlinear multi-agent system, where the time of synchronization can be preassigned. Developed control can be used to synchronize large-scale, higher-order multi-agent systems as passivity is an important property in designing control for complex control systems, where the control inputs and outputs are considered in determining the stability of the system in contrast to other approaches, such as state-based Control We introduced the notion of predefined-time passivity and as an application of the exposed stability analysis, static and adaptive predefined-time control algorithms are designed to study the average consensus problem for nonlinear leaderless multiagent systems in predefined-time. We provide a detailed mathematical analysis of the proposed protocol, including convergence proof and stability analysis. We discussed the tracking problem for a single agent, and designed state feedback and adaptive state feedback control scheme to make tracking error predefined-time passive and then showed that in the absence of external input, tracking error reduces to zero in predefined-time. Furthermore, we extended this concept for a nonlinear multi-agent system and designed state feedback and adaptive state feedback control scheme which ensure synchronization of all the agents in predefined-time. To further strengthen the idea, we applied our control scheme to a nonlinear multi-agent system by taking the example of Chua’s circuit. Finally, we compared the result of our developed predefined-time synchronization framework with finite-time synchronization scheme available in literature for the Kuramoto model.

## 1. Introduction

A multi-agent system (MAS) is a complex system made up of several agents collaborating to accomplish a single objective. The synchronization problem of MAS has drawn the attention of many researchers in past few years due to its possible application in various areas such as the cooperation of unmanned air vehicles [1], mobile robots [2], cooperative attack of missiles [3], formation of satellites [4], exploration [5], surveillance and rescue tasks [6,7,8,9] among others. Additionally, in various literature, MAS has found possible application in the formation of satellites [4], air traffic control [10], flocking [11,12], and rendezvous [13,14]. These applications can be broadly grouped based on the nature of the problem being investigated under consensus, trajectory tracking, formation control, coordination, or synchronization [15,16,17,18,19]. MAS synchronization can be analyzed broadly into two categories: leaderless MAS synchronization and leader-follower MAS synchronization. In this paper, we have followed leaderless MAS synchronization, where the final consensus is dependent on the initial conditions of all the agents whereas in leader-follower MAS synchronization, the final consensus depends upon the state of the leader.

Synchronization is a widely investigated problem in nonlinear multi-agent systems. In [20] the author addressed the synchronous control of homogeneous autonomous linear systems, as in the case of an underwater robot swarm. In [21], the author presented the autonomous organization of an aerial robot swarm. In [22], the authors investigated the path planning and control cluster of unmanned aerial vehicles under the hazardous environmental situation. In [23], the authors proposed the synchronous motion of a two-group aerial swarm using particle swarm optimization. Su et al. [24] considered MAS as having unknown nonlinearities and using distributed control scheme. In [25], the authors addressed synchronization problems that involve nonlinear couplings among agents and proposed an event-triggered control scheme to mitigate them. In [26], authors investigated the consensus problem of MAS with nonlinear controller output using event-triggered control for digital communication networks. Similarly, in [27] the authors considered the problem of MAS with periodic event-triggered synchronization for linear systems in the presence of communication delay. In [28], the authors considered discrete-time MAS having network topology with varying time delay, and two different synchronization criteria were investigated. In the same paper, two types of communication networks are investigated for state synchronization: full-state coupling and partial-state coupling. In paper [29], the authors considered homogenous MAS having partial state coupling and solvability conditions were derived considering directed and weighted network topology. In paper [30] the author has arrived at necessary and sufficient condition for consensusability of linear MAS.

Convergence speed and the actual time of convergence are very important parameters for MAS synchronization. In all the above-mentioned literature, the formulated problem was to synchronize agents asymptotically. However, for all practical purposes, it is expected that agents’ synchronization should take place in finite time, which motivated researchers to explore the possibility of agents synchronization in finite time. In papers [31,32,33], finite-time consensus algorithms were proposed for first-order MAS. In [34], authors have formulated the problem of MAS having second-order dynamics, where agents’ synchronization is achieved using output feedback control. In [35], authors discussed finite time synchronization problems for a nonlinear MAS, with uncertainties and delay. In paper [36], authors studied the finite-time consensus problem of MAS having disturbance, and using the Hölder Lyapunov function, sufficient conditions were derived for finite-time consensus. In paper [37], authors investigated the finite-time consensus of nonlinear MAS under communication constraints, where a distributed discontinuous control algorithm was proposed. Other works for the realization of finite-time synchronization of MAS were investigated in [38,39,40,41,42,43,44,45].

As per the notion of passivity, if a system is passive, then it possesses stability in the absence of any external input. In [46], passivity theory was first used for circuit analysis. Later, passivity was successfully applied to stability [47], chaos control, synchronization [48]. It was found that passivity is a useful tool to investigate the stabilization [49] and tracking of nonlinear dynamical systems. In the passivity framework, we describe the stored energy of the system using storage function. The storage function can also be used as a Lyapunov candidate, if the external input to the system is considered to be zero. Passivity has an elegant feature that it is preserved under state feedback and parallel interconnections. Hence, the passivity framework is a suitable tool to stabilize large-scale interconnected systems, such as MAS.

The passive system has an elegant property that the stored energy of the system goes to zero as time goes to infinity, in the absence of an external supply. However, for many practical purposes, it is expected that the stored energy should go to zero in some finite time. In [39], the author investigated the attitude control problem of a rigid body using the finite-time control notion which is based on the passivity property of a linear system. Also, the same authors have laid down the framework of finite-time passivity. Later, in [40], the authors redefined the notion of finite-time passivity, and it was shown that if two passive systems are joined in feedback or in parallel, the combined system remains passive. This fact was further used for the development of the passivity framework for MAS in connection with finite-time control theory to develop the notion of finite-time passivity (FTP). Further, in [50,51], the authors used the FTP notion to solve the finite-time synchronization problem of multi-agent systems.

In the case of FTP, the prime limitation was that the convergence time is governed by the system’s initial conditions, resulting in different convergence times for different initial conditions, which sets restrictions on its real-time applications as, for major practical purposes, the system’s initial condition used to be unknown. fixed time stability [52]. In further extension to this, fixed-time stability was proposed, where the settling time function is the upper bound of all the convergence times, and it is independent of its initial conditions. In [53,54], authors have used the fixed-time stability notion to synchronize MAS. In [55], the notion of fixed-time passivity (FXTP) is used for interconnected memristive neural networks. For the case of fixed-time passive systems, the convergence time depends on the system’s parameters. The above-stated restrictions were addressed by using the notion of predefined-time stability [56,57] and the prescribed finite time notion [58]. In the paper [58], the authors successfully overcame the constraints of fixed-time stability by assuring the settling time function is independent of initial conditions and other design parameters. However, in paper [56], a new method for designing controllers is proposed, where the time of convergence can be fixed a priori and is independent of initial conditions. The idea of predefined-time stability [56] find its use in various real-life applications [59,60].

In the present paper, we have combined the notion of predefined-time stability [56] with passivity to develop a predefined-time passivity framework. The developed framework is later used to synchronize trajectories of nonlinear MAS, where we are synchronizing agents of nonlinear MAS in a predefined time, specified a priori. Earlier work on predefined-time convergence for MAS is based on sliding mode control [61], however, we have used a passivity framework, which can be used to synchronize large-scale multi-agent system as passivity is an important property in designing control for complex control systems, it remains preserved under feedback and parallel interconnection. In passivity based control, the control inputs, and outputs are considered to be the most important variables in determining the stability of the system. This is in contrast to other approaches, such as state-based control, where the internal states of the system are the primary focus. Also, our proposed control is applicable to higher order MAS, as presented in the example of Chua’s circuit, whereas in [61], predefined-time synchronization is discussed for second-order systems. Additionally, the proposed control scheme provides the exact time of convergence of the agents, which can be chosen a priori. Hence, the predefined-time passivity framework allows the convergence time to be chosen a priori for large-scale multi-agent systems, which can be useful in many practical applications where the desired convergence time may vary.

Firstly, we have developed a tracking problem for a single agent, using a framework of predefined-time passivity and designed control laws as state feedback and adaptive state feedback to make error dynamics to be predefined-time passive. Also, in the absence of external input we have shown that tracking error reduces to zero in a predefined time. Later we designed state feedback and adaptive state feedback control laws for nonlinear MAS for synchronization at a predefined time. To further demonstrate the findings, we have shown an example of Chua’s circuit. We have shown that synchronization of agents with respect to each other occurs at a predefined time, which is specified a priori.

The further part of the paper goes ahead as follows. The mathematical notions and preliminary results are mentioned in Section 2. Section 3 provides the main results. Examples with simulation results are illustrated in Section 4. Finally, a brief conclusion ends the paper.

## 2. Methods and Materials

In this section, we will discuss those terminologies and notions important for the development of the rest of the paper. R is used to denote the set of real numbers and $R_{+}$ denotes the set of non-negative real numbers. $R^{n}$ represents *n*-tuple vector. To represent the least eigenvalue of a matrix *C*, $\lambda_{s}\left(C\right)$ is used. *I* represents the identity matrix.

### 2.1. Graph Theory

Some graph theory [62] notions are now discussed. We represent multi-agent system by a graph O=(U,D,J). Here, the vertices of the graph is represented by $U=\{v_{1},v_{2}\cdots ,v_{n}\}$. The set of edges is denoted by D⊆U×U. $J={\left[a_{ij}\right]}_{n\times n}$ is the adjacency matrix satisfying $a_{ij}=1$ if $v_{i},v_{j}\in D$ else $a_{ij}=0$. We represent the degree matrix as $B=\mathrm{diag}\left(b\left(\eta_{i}\right)\right)\in R^{n\times n}$, where $b(\eta_{i})$ denotes number of nodes linked to node $\eta_{i}$. For the graph O, the Laplacian matrix satisfies the relation $P=J-B\in R^{n\times n}$.

### 2.2. K-Class Function

A strictly increasing and continuous function $g:R_{+}\to R_{+}$ with g(0)=0 is called as *K*-class function [63].

### 2.3. Kronecker Product

The Kronecker product is denoted by the symbol ⊗. If *X* is a p×q matrix and *Y* is an l×n matrix, then X⊗Y gives the lp×nq block matrix.

We use the following two systems for the purpose of validation of our proposed controller.

### 2.4. Chua’s Circuit

Chua’s circuit is one of the simplest electronic circuits that exhibits chaotic behavior and real-world applications typically use synchronized chaotic circuits. We consider a nonlinear MAS consisting of four Chua’s circuits where each circuit’s dynamics is given by

(1) $$\left[\begin{array}{c} {\dot{\eta }}_{k1}\left(t\right)\\ {\dot{\eta }}_{k2}\left(t\right)\\ {\dot{\eta }}_{k3}\left(t\right) \end{array}\right]=\left[\begin{array}{c} 10(-\eta_{k1}\left(t\right)+\eta_{k2}\left(t\right)+g\left(\eta_{k1}\left(t\right)\right)\\ \eta_{k1}\left(t\right)-2\eta_{k2}\left(t\right)+\eta_{k3}\left(t\right)\\ -14.87\eta_{k3}\left(t\right) \end{array}\right]+\mu_{k}\left(t\right)$$

for k=1,2,3,4, where $\eta_{k}={\left[\eta_{k1},\eta_{k2},\eta_{k3}\right]}^{⊤}$ is the state vector and $\mu_{k}={\left[\mu_{k1},\mu_{k2},\mu_{k3}\right]}^{⊤}$ is the control vector, $y_{k}\left(t\right)=\eta_{k}\left(t\right)={\left[y_{k1},y_{k2},y_{k3}\right]}^{⊤}$ is the output vector and $g\left(\eta_{k1}\left(t\right)\right)=-0.68\eta_{k1}\left(t\right)+0.5\left(-1.27+0.68\right)\left(|\eta_{k1}\left(t\right)+1\right)\left|-\right|\eta_{k1}\left(t\right)-1\left|\right)$.

### 2.5. Kuramoto Model

Another important phase oscillator is the Kuramoto model, where each oscillator has its own intrinsic natural frequency. We have considered 6 such oscillators with the following dynamics of each oscillator.

(2) $$\begin{array}{c} \begin{array}{cc} {\dot{\eta }}_{k}\left(t\right) & =\omega +\frac{A}{6}\sum_{q=1}^{6}sin\left(\eta_{q}\left(t\right)-\eta_{k}\left(t\right)\right)+\mu_{k}\left(t\right)\\ y_{k}\left(t\right) & =\eta_{k}\left(t\right) \end{array} \end{array}$$

where k=1,⋯,6, the phase of the *k*-th oscillator is represented by $\eta_{k}\in R$, oscillators have the natural frequency ω and *A* denotes the coupling gain. $\mu_{k}$ and $y_{k}$ denotes the input and output respectively, of the agent *k*.

The following definitions are needed for the further development Predefined-time passivity framework.

Refer to the following forced system

(3) $$\dot{\eta }=G\left(t,\eta ,\mu ,\rho \right),\;\;\;\eta \left(t_{0}\right)=\eta_{0}\in M\subset R^{n}$$

here $\eta \in M\subset R^{n}$ denotes the states, $\rho \in R^{k}$ is the system parameters, the control input is $\mu \in R^{m}$, $G:R_{+}\times M\times R^{m}\times R^{}\to R^{n}$ is a nonlinear function such that F(t,0,0,ρ)=0, i.e., the equilibrium point of (3) is η(t)=0, and $t_{0}\in R_{+}$ denotes the initial time.

**Definition** **1 ([64]).** *The system (3) is said to be FTS (Finite Time Stable) about the origin if*

- $\eta (t,t_{0},\eta_{0},\mu ,\rho )=0$ *is asymptotically stable, and*
- $\eta (t,t_{0},\eta_{0},\mu ,\rho )=0$ ∀ $t\geq t_{0}+T\left(t_{0},\eta_{0},\mu ,\rho \right)$*, with* $T:R_{+}\times M\times R^{m}\times R^{k}\to R_{+}$ *as the time of convergence*.

**Definition** **2 ([56,58]).** *The system (3) with control* $\mu :=\mu \left(t,\eta ,t_{F},c\right),\;t_{F}\in R_{+},c\in R,$ *is said to be predefined-time stable about the origin if*

- *it is finite-time stable,*
- *there exists time* $t_{F}>0,c>n$*, which is independent of any initial conditions and system parameters and can be chosen a priori, and*
- *$t_{F}\geq t_{A}$* ∀ $\eta_{0}\in M$*, where* $t_{A}$ *denotes the actual time of convergence to the origin of the system’s state trajectories.*

*The origin of system (3) is said to be globally predefined-time stable if* $M=R^{n}$.

Next, consider a time-varying dynamical system

$$\begin{array}{cc} \dot{\eta }=-\psi \left(t,\eta \right):= & \left\{\begin{array}{c} \frac{-c(e^{\eta }-1)}{e^{\eta }\left(\tau_{f}-t\right)},\;\;\;\text{if}\;t\in \left[t_{0},\tau_{f}\right)\\ 0,\;\;\;\;\;\;\;\;\;\;\;\text{otherwise} \end{array}\right. \end{array}$$

where η∈R denotes the state, c>1, $t_{0}\in R_{+}$ denotes the initial time such that $\tau_{f}=t_{F}+t_{0}$, with $t_{F}$ as the predefined-time. Above differential equation denotes the predefined-time dynamics as both η(t) and $\dot{\eta }\left(t\right)$ are zero for all $t\geq \tau_{f}$.

**Lemma** **1 ([56]).** *Take the system (3) with a domain* $M\subset R^{n}$ *having the origin. Assume* $f_{1}\left(\eta \right)$ *and* $f_{2}\left(\eta \right)$ *be two continuous positive definite functions on* M*. If there exist a continuously differentiable function* $W:I_{s}\times M\to R_{+}$ *(*$I_{s}=\left[t_{0},\infty \right)$*) and* c>1 *:*

- $f_{1}\left(\eta \right)\leq W\left(t,\eta \right)\leq f_{2}\left(\eta \right),\;\;\forall \;t\in I_{s},\;\forall \;\eta \in M\setminus \left\{0\right\}$
- $W\left(t,0\right)=0,\;\;\forall \;t\in I_{s}\;$
- $\dot{W}\left(t,\eta \right)\leq \left\{\begin{array}{c} \frac{-c(e^{W(t,\eta )}-1)}{e^{W(t,\eta )}\left(\tau_{f}-t\right)},\;\;\;\;\;\text{if}\;\;t\in \left[t_{0},\tau_{f}\right)\\ 0,\;\;\;\;\;\;\;\;\;\;\;\;\;\;\;\;\;\;\;\text{otherwise} \end{array}\right.$

*for* W≠0*, then the origin will be predefined-time stable and* $t_{F}=\tau_{f}-t_{0}\geq t_{A}$*. If* $M=R^{n}$ *and W is radially unbounded, then* η(t)=0 *is said to be globally predefined-time stable. As already mentioned in [63], in context of passivity, the energy dissipation guarantees the closed-loop stability irrespective of the system nonlinearities, and therefore we provide here the notion of passivity in order to establish our main results.*

*Refer to the following forced system*

(4) $$X:\left\{\begin{array}{c} \dot{\eta }=G\left(\eta ,\zeta \right),\;\;\;\eta \left(t_{0}\right)=\eta_{0}\in R^{n}\\ y=H(\eta ,\zeta ) \end{array}\right.$$

*here* $\eta \in R^{n}$ *denotes the state vector, the external input is* $\zeta \in R^{k}$ *and* $y\in R^{k}$ *denotes the output of the system.* $G:R^{n}\times R^{k}\to R^{n}$ *is assumed to be locally Lipschitz and* $H:R^{n}\times R^{k}\to R^{k}$ *is considered to be a continuous function.*

**Definition** **3 ([63]).** *A system* X *having output* $y\in R^{k}$ *and external input* $\zeta \in R^{k}$ *is called passive if there exists a smooth function* $V:R^{n}\to R_{+}$ *satisfying:* $\dot{V}\leq \zeta^{⊤}y$.

## 3. Results and Findings

Here we introduce the notion of predefined-time passivity and subsequently utilize it for agents’ synchronization in predefined-time.

### 3.1. Predefined-Time Passivity

Refer to the following forced system

(5) $$\Delta :\;\;\left\{\begin{array}{c} \dot{\eta }=G\left(t,\eta ,\zeta \right),\;\;\;\eta \left(t_{0}\right)=\eta_{0}\in M\subset R^{n}\\ y=H(t,\eta ,\zeta ), \end{array}\right.$$

with $\eta \in M\subset R^{n}$ as the states, $\zeta \in R^{k}$ be the external input, and $y\in R^{k}$ as the output. $G:R_{+}\times M\times R^{k}\to R^{n}$ is a locally Lipschitz nonlinear function and $H:R_{+}\times M\times R^{k}\to R^{k}$ is a nonlinear continuous function. Assuming (t,0,0) as the equilibrium point of system (5), the notion of predefined-time passivity is given below.

**Definition** **4.** *System* Δ *having output* $y\in R^{k}$ *and external input* $\zeta \in R^{k}$ *is said to be passive in predefined-time when* ∃ *a positive smooth function* $V:M\to R_{+}$:

(6) $$\dot{V}\leq \zeta^{⊤}y-\frac{cg\left(V\right)\left(e^{g(V)}-1\right)}{e^{g(V)}\left(\tau_{f}-t\right)}\;\;\;\;\text{for}\;t\in \left[t_{0},\tau_{f}\right)$$

*and*

(7) $$\dot{V}\leq \zeta^{⊤}y\;\;\;\;\;\text{for}\;t>\tau_{f},$$

*where* $\tau_{f}=t_{F}+t_{0}$*, with* $t_{F}$ *as the predefined-time,* c>1 *and* g(·) *is a class K function.*

**Remark** **1.** *There is a close relationship between stability and the passivity-based framework. Lyapunov function can be chosen as a candidate for a storage function in passive systems. Along with stability, there is an interesting property associated with passivity for MAS. If 2 passive systems are joined in feedback/parallel, the overall system remains passive, this passivity preservation property allows component-wise analysis of the complex large-scale system which reduces effort in designing and analyzing large-scale systems like MAS. This is the motivation to develop a passivity-based framework for MAS in combination with a predefined-time notion.*

**Remark** **2.** *Finite-time notion and passivity framework together as a finite-time passivity has been exploited for many years for synchronization of nonlinear MAS [50,65]. From all practical viewpoints, for the synchronization problem, the time of convergence is crucial, and it is beneficial if the time of convergence can be fixed a priori which is the case of predefined-time passivity. Also, in the case of predefined-time passivity, convergence time is independent of initial conditions and system parameters. This encourages us to investigate and use the idea of predefined-time passivity for nonlinear MAS.*

### 3.2. Predefined-Time Passivity along with Tracking of Single Agent in Predefined-Time

Consider a nonlinear MAS with *L* agents. Each agent has the following dynamics:

(8) $$\begin{array}{c} {\dot{\eta }}_{k}\left(t\right)=\phi \left(\eta_{k}\left(t\right)\right)+\mu_{k}\left(t\right),\;\;\;\;\;\;y_{k}\left(t\right)=\eta_{k}\left(t\right) \end{array}$$

for k=1,⋯,L, with ϕ(·) as the nonlinear function, $\eta_{k}\left(t\right)={\left[\eta_{k1}\left(t\right),\eta_{k2}\left(t\right),\cdots ,\eta_{kn}\left(t\right)\right]}^{⊤}\in M\subset R^{n}$ denotes the state vector for the k-th agent. $\mu_{k}\left(t\right)={\left[\mu_{k1}\left(t\right),\mu_{k2}\left(t\right),\cdots ,\mu_{kn}\left(t\right)\right]}^{⊤}\in R^{n}$ is the control input and $y_{k}\left(t\right)={\left[y_{k1}\left(t\right),y_{k2}\left(t\right),\cdots ,y_{kn}\left(t\right)\right]}^{⊤}\in R^{n}$ is the output for the *k*-th agent. Considering function ϕ(·) to be Lipschitz i.e.,

(9) $$∥\phi \left(x_{1}\right)-\phi \left(x_{2}\right)∥\leq l∥x_{1}-x_{2}∥$$

for $x_{1},x_{2}\in M\subset R^{n},\;0<l\in R$. Suppose a reference trajectory

${\bar{\eta }}_{k}\left(t\right)={\left[{\bar{\eta }}_{k1}\left(t\right),{\bar{\eta }}_{k2}\left(t\right),\cdots ,{\bar{\eta }}_{kn}\left(t\right)\right]}^{⊤}\in M\subset R^{n}$ which is the solution to the system below.

(10) $$\begin{array}{c} {\dot{\bar{\eta }}}_{k}\left(t\right)=\phi \left({\bar{\eta }}_{k}\left(t\right)\right) \end{array}$$

Taking $r_{k}\left(t\right)=\eta_{k}\left(t\right)-{\bar{\eta }}_{k}\left(t\right)={\left[r_{k1}\left(t\right),r_{k2}\left(t\right),\cdots ,r_{kn}\left(t\right)\right]}^{⊤}\in M\subset R^{n}$ as the vector of tracking error, then:

(11) $$\begin{array}{c} \begin{array}{cc} {\dot{r}}_{k}\left(t\right)= & \phi \left(\eta_{k}\left(t\right)\right)-\phi \left({\bar{\eta }}_{k}\left(t\right)\right)+\mu_{k}\left(t\right)\\ y_{k}^{\prime }\left(t\right)= & r_{k}\left(t\right) \end{array} \end{array}$$

for k=1,⋯,L, with $y_{k}^{\prime }\left(t\right)=y_{k}\left(t\right)-{\bar{\eta }}_{k}\left(t\right)$. Now, to establish error dynamics (11) to be predefined-time passive (PTP) using the developed controller, we present the following result.

**Theorem** **1.** *Refer to the system (11) and let the developed state-feedback control be*

(12) $$\begin{array}{cc} \mu_{k}\left(t\right)= & \left\{\begin{array}{c} \zeta_{k}\left(t\right)-Sr_{k}\left(t\right)-\psi_{k}\left(t,r_{k}\left(t\right)\right),\text{if}\;t\in \left[t_{0},\tau_{f}\right)\\ \zeta_{k}\left(t\right)-Sr_{k}\left(t\right),\;\;\;\;\;\;\;\;\;\;\;\;\;\;\;\;\text{otherwise} \end{array}\right. \end{array}$$

*here $\zeta_{k}\left(t\right)={\left[\zeta_{k1}\left(t\right),\zeta_{k2}\left(t\right),\cdots ,\zeta_{kn}\left(t\right)\right]}^{⊤}\in R^{n}$ is the external input to the agent k, $\psi_{k}\left(t,r_{k}\left(t\right)\right)={\left[\frac{c(e^{r_{k1}\left(t\right)}-1)}{e^{r_{k1}\left(t\right)}\left(\tau_{f}-t\right)},\frac{c(e^{r_{k2}\left(t\right)}-1)}{e^{r_{k2}\left(t\right)}\left(\tau_{f}-t\right)},\cdots ,\frac{c(e^{r_{kn}\left(t\right)}-1)}{e^{r_{kn}\left(t\right)}\left(\tau_{f}-t\right)}\right]}^{⊤}$, 0<S∈R and c>1. If S>l, where 0<l∈R, then, the system (11) achieves predefined-time passivity.*

**Proof.**  Let’s take a following storage function

(13) $$\begin{array}{c} V=\frac{1}{2}r_{k}^{⊤}\left(t\right)r_{k}\left(t\right) \end{array}$$

We give derivative of V along the trajectories of system (11) for $t\in [t_{0},\tau_{f})$ by

(14)V˙=rk⊤(t)r˙k(t)(15)=rk⊤(t)(ϕ(ηk(t))−ϕ(η¯k(t))+μk(t))=rk⊤(t)(ϕ(ηk(t))−ϕ(η¯k(t)))+rk⊤(t)ζk(t)−Srk⊤(t)rk(t)

(16) $$\begin{array}{cc} \; & \;-c\sum_{q=1}^{n}r_{kq}\left(t\right)\frac{\left(e^{r_{kq}\left(t\right)}-1\right)}{e^{r_{kq}\left(t\right)}\left(\tau_{f}-t\right)}. \end{array}$$

Since ϕ(·) is assumed to be Lipschitz function,

(17) $$r_{k}^{⊤}\left(\phi \left(\eta_{k}\left(t\right)\right)-\phi \left({\bar{\eta }}_{k}\left(t\right)\right)\right)\leq lr_{k}^{⊤}\left(\eta_{k}\left(t\right)-{\bar{\eta }}_{k}\left(t\right)\right)$$

Using (13), we can write

$$V\geq \frac{1}{2}r_{k1}^{2}\Rightarrow \sqrt{2V}\geq r_{k1},V\geq \frac{1}{2}r_{k2}^{2}\Rightarrow \sqrt{2V}\geq r_{k2}$$

Similarly,

$$V\geq \frac{1}{2}r_{k3}^{2}\Rightarrow \sqrt{2V}\geq r_{k3},\cdots ,\sqrt{2V}\geq r_{kn}$$

Then, $\dot{V}$ becomes

(18) $$\begin{array}{c} \begin{array}{cc} \dot{V}\leq & \left(l-S\right)r_{k}^{⊤}r_{k}-c\sum_{q=1}^{n}\left|r_{kq}\right|\frac{\left(e^{|r_{kq}|}-1\right)}{e^{|r_{kq}|}\left(\tau_{f}-t\right)}+r_{k}^{⊤}\zeta_{k} \end{array} \end{array}$$

As $V=\frac{1}{2}\left(r_{k1}^{2}+r_{k2}^{2}+\cdots ,r_{kn}^{2}\right)$, then $V\leq \frac{n}{2}\left(\max\{\right|r_{k1}\left|,\right|r_{k2}\left|,\cdots ,\right|r_{kn}{\left|\}\right)}^{2}$. At a particular instant of time, the max function produces one variable, suppose it gives $|r_{k1}|$, then $\sqrt{\frac{2V}{n}}\leq \left|r_{k1}\right|$. Then,

(19) $$-c|r_{k1}|\frac{\left(e^{|r_{k1}|}-1\right)}{e^{|r_{k1}|}\left(\tau_{f}-t\right)}\leq -c\sqrt{\frac{2V}{n}}\frac{\left(e^{\sqrt{\frac{2V}{n}}}-1\right)}{e^{\sqrt{\frac{2V}{n}}}\left(\tau_{f}-t\right)}$$

Now, (18) becomes

(20) $$\begin{array}{c} \dot{V}\leq \left(l-S\right)r_{k}^{⊤}r_{k}-c\sqrt{\frac{2V}{n}}\frac{\left(e^{\sqrt{\frac{2V}{n}}}-1\right)}{e^{\sqrt{\frac{S}{n}}}\left(\tau_{f}-t\right)}+r_{k}^{⊤}\zeta_{k} \end{array}$$

When S>l, then

(21) $$\begin{array}{c} \dot{V}\leq -c\sqrt{\frac{2V}{n}}\frac{\left(e^{\sqrt{\frac{2V}{n}}}-1\right)}{e^{\sqrt{\frac{2V}{n}}}\left(\tau_{f}-t\right)}+r_{k}^{⊤}\zeta_{k} \end{array}$$

After $t>\tau_{f}$, one can observe that with the proposed control (12) the $\dot{V}$ becomes

(22) $$\begin{array}{c} \dot{V}\leq r_{k}^{⊤}\zeta_{k} \end{array}$$

Hence, the system (11) becomes PTP under the output $r_{k}$ and external input $\zeta_{k}$, with $g\left(V\right)=\sqrt{\frac{2V}{n}}$, and c>1. □

**Corollary** **1.** *For the system (11) consider the control (12) with $\zeta_{k}\left(t\right)=0$, then we say the origin of the dynamics of the error (11) to be stable in predefined-time.*

**Proof.**  The proof is analogous to Theorem 1 but with $\zeta_{k}\left(t\right)=0$. In that case, for $t\in [t_{0},\tau_{f})$ the inequality (21) becomes

(23) $$\begin{array}{c} \begin{array}{cc} \dot{V}\leq & -c\sqrt{\frac{2V}{n}}\frac{\left(e^{\sqrt{\frac{2V}{n}}}-1\right)}{e^{\sqrt{\frac{2V}{n}}}\left(\tau_{f}-t\right)} \end{array} \end{array}$$

Let $\Lambda =\sqrt{\frac{2V}{n}}$, then $\dot{\Lambda }=\frac{\dot{V}}{\sqrt{n}\sqrt{2V}}$, thus the above dynamics (23) becomes the dynamics of predefined time, i.e.,

(24) $$\dot{\Lambda }=-c^{\prime }\frac{\left(e^{\Lambda }-1\right)}{e^{\Lambda }\left(\tau_{f}-t\right)}$$

here $c^{\prime }=\frac{c}{n}$, and for $t>\tau_{f}$, $\dot{V}\leq 0$. Therefore, from Lemma 1, the error (11) reaches zero in the predefined time. In addition, in order to get rid-off from the Lipschitzness condition of the function ϕ(·), following result is given using the developed adaptive state-feedback control. □

**Theorem** **2.** *Consider the dynamics (11). Let the adaptive control be*

(25) $$\begin{array}{cc} \mu_{k}\left(t\right)= & \left\{\begin{array}{c} \zeta_{k}\left(t\right)-S\left(t\right)r_{k}\left(t\right)-\psi_{k}\left(t,r_{k}\left(t\right)\right),\;\text{if}\;t\in \left[t_{0},\tau_{f}\right)\\ \zeta_{k}\left(t\right)-S\left(t\right)r_{k},\;\;\;\;\;\;\;\;\;\;\;\;\;\;\;\;\;\;\;\;\;\;\;\;\text{otherwise} \end{array}\right. \end{array}$$

*and*

(26) $$\begin{array}{c} \dot{S}\left(t\right)=ar_{k}^{⊤}\left(t\right)r_{k}\left(t\right)+b \end{array}$$

*where all the variables remain the same as in (12), a>0∈R, b>0∈R and 0<S(0)∈R. Then using the control (25), the system (11) can achieve predefined-time passivity.*

**Proof.**  From (18), one can write $\dot{V}$ along the trajectories of system (11) for $t\in [t_{0},\tau_{f})$ as

(27) $$\begin{array}{c} \begin{array}{cc} \dot{V}\leq & \left(l-S\left(t\right)\right)r_{k}^{⊤}r_{k}-c\sum_{q=1}^{n}\left|r_{kq}\right|\frac{\left(e^{|r_{kq}|}-1\right)}{e^{|r_{kq}|}\left(\tau_{f}-t\right)}+r_{k}^{⊤}\zeta_{k} \end{array} \end{array}$$

From (26), one can find a $0<t_{0}\in R$ satisfying S(t)≥l for all $t\geq t_{0}$.

Hence, for $t_{0}\leq t<\tau_{f}$, we have

(28) $$\begin{array}{c} \dot{V}\leq -c\sqrt{\frac{2V}{n}}\frac{\left(e^{\sqrt{\frac{2V}{n}}}-1\right)}{e^{\sqrt{\frac{2V}{n}}}\left(\tau_{f}-t\right)}+r_{k}^{⊤}\zeta_{k} \end{array}$$

After time $t>\tau_{f}$, it is evident that dynamics of $\dot{V}$ is,

(29) $$\begin{array}{c} \dot{V}\leq r_{k}^{⊤}\zeta_{k} \end{array}$$

with the proposed control (25). Hence, the system (11) is said to be PTP using (28) and (29), considering $r_{k}$ and $\zeta_{k}$ as the output and external input, respectively, to the *k*-th agent, $g\left(V\right)=\sqrt{\frac{2V}{n}}$, and c>1. □

In a similar way as proved in Corollary 1, the origin of the dynamics of tracking error (11) is predefined-time stable with the controller (25) if the external input $\zeta_{k}\left(t\right)=0$.

### 3.3. Predefined Time Synchronization of MAS Using Passivity

**Definition** **5.** *The MAS (8) undergoes synchronization in predefined-time if*

(30) $$∥\eta_{k}\left(t\right)-\frac{1}{L}\sum_{r=1}^{L}\eta_{r}\left(t\right)∥=0\;\;\mathrm{in}\;t\leq \tau_{f}$$

*and $\tau_{f}$ is the predefined time.*

*Let us define the error as: ${\tilde{r}}_{k}\left(t\right)=\eta_{k}\left(t\right)-\bar{\eta }\left(t\right)={\left[{\tilde{r}}_{k1}\left(t\right),{\tilde{r}}_{k2}\left(t\right)\cdots ,{\tilde{r}}_{kn}\left(t\right)\right]}^{⊤}\in N\subset R^{n}$, and $\bar{\eta }\left(t\right)=\frac{1}{L}\sum_{r=1}^{L}\eta_{r}\left(t\right)$. Error dynamics can be written as*

(31) $$\begin{array}{c} \begin{array}{cc} {\dot{\tilde{r}}}_{k}\left(t\right)= & \;\phi \left(\eta_{k}\left(t\right)\right)-\frac{1}{L}\sum_{r=1}^{L}\phi \left(\eta_{r}\left(t\right)\right)+\mu_{k}\left(t\right)-\frac{1}{L}\sum_{r=1}^{L}\mu_{r}\left(t\right)\\ {\tilde{y}}_{k}\left(t\right)= & \;{\tilde{r}}_{k}\left(t\right) \end{array} \end{array}$$

*for k=1,⋯,L, and ${\tilde{y}}_{k}\left(t\right)=y_{k}\left(t\right)-\bar{\eta }\left(t\right)$.*

**Theorem** **3.** *Consider the MAS (8) having the following coupling control*

(32) $$\begin{array}{cc} \mu_{k}= & \left\{\begin{array}{c} \zeta_{k}+\sum_{q=1}^{L}P_{kq}Q{\tilde{r}}_{k}-S{\tilde{r}}_{k}-\psi_{k}\left(t,{\tilde{r}}_{k}\right),\;\;\text{if}\;t\in \left[t_{0},\tau_{f}\right)\\ \zeta_{k}+\sum_{q=1}^{L}P_{kq}Q{\tilde{r}}_{k}-S{\tilde{r}}_{k},\;\;\;\;\;\;\;\;\;\;\;\;\;\;\;\;\;\text{otherwise} \end{array}\right. \end{array}$$

*where $\zeta_{k}\left(t\right)={\left[\zeta_{k1}\left(t\right),\zeta_{k2}\left(t\right),\cdots ,\zeta_{kn}\left(t\right)\right]}^{⊤}\in R^{n}$ is the external input to the agent k, $\psi_{k}\left(t,{\tilde{r}}_{k}\left(t\right)\right)={\left[\frac{c(e^{{\tilde{r}}_{k1}\left(t\right)}-1)}{e^{{\tilde{r}}_{k1}\left(t\right)}\left(\tau_{f}-t\right)},\frac{c(e^{{\tilde{r}}_{k2}\left(t\right)}-1)}{e^{{\tilde{r}}_{k2}\left(t\right)}\left(\tau_{f}-t\right)},\cdots ,\frac{c(e^{{\tilde{r}}_{kn}\left(t\right)}-1)}{e^{{\tilde{r}}_{kn}\left(t\right)}\left(\tau_{f}-t\right)}\right]}^{⊤},c>1$, 0<S∈R, $P_{kq}\in R^{L\times L}$ is Laplacian of graph O defined such that: $P_{kq}=P_{qk}\in R>0$ when an edge exists between agent k and q (q≠k), else $P_{kq}=P_{qk}=0\;\left(q\neq k\right)$, and*

(33) $$P_{kk}=-\sum_{q=1\;q\neq k}^{L}P_{kq}$$

*and further $Q\in R^{n\times n}>0$. Hence, the MAS (8) is PTP whose agents undergo synchronization in predefined-time if ζ(t)=0, with $\zeta \left(t\right)={\left[\zeta_{1}^{⊤}\left(t\right),\zeta_{2}^{⊤}\left(t\right),\cdots ,\zeta_{L}^{⊤}\left(t\right)\right]}^{⊤}$.*

**Proof.**  Consider the storage function as

(34) $$\begin{array}{c} V=\frac{1}{2}\sum_{k=1}^{L}{\tilde{r}}_{k}^{⊤}\left(t\right){\tilde{r}}_{k}\left(t\right) \end{array}$$

Thus, the derivative of V along the system trajectories (31) for $t\in [t_{0},\tau_{f})$ can be written as

(35)V˙=∑k=1Lr˜k⊤(t)r˜˙k(t)=∑k=1Lr˜k⊤(t)(ϕ(ηk(t))−ϕ(η¯(t))+ϕ(η¯(t))+ζk(t)−1L∑r=1Lϕ(ηr(t))+∑q=1LPkqQr˜k(t)−Sr˜k(t)(36)−ψk(t,r˜k(t))−1L∑r=1Lμr(t))

Since

(37) $$\begin{array}{c} \begin{array}{cc} \sum_{k=1}^{L}{\tilde{r}}_{k}^{⊤}\left(t\right)\left(\phi \left(\bar{\eta }\left(t\right)\right)-\frac{1}{L}\sum_{r=1}^{L}\phi \left(\eta_{r}\left(t\right)\right)\right)= & \;0\\ \sum_{k=1}^{L}{\tilde{r}}_{k}^{⊤}\left(t\right)\left(\frac{1}{L}\sum_{r=1}^{L}\mu_{r}\left(t\right)\right)= & \;0 \end{array} \end{array}$$

Using the Lipschitzness of ϕ(·), we write

(38) $$\begin{array}{c} {\tilde{r}}_{k}^{⊤}\left(t\right)\left(\phi \left(\eta_{k}\left(t\right)\right)-\phi \left(\bar{\eta }\left(t\right)\right)\right)\leq S{\tilde{r}}_{k}^{⊤}\left(t\right){\tilde{r}}_{k}\left(t\right),0<S\in R \end{array}$$

Incorporating (37) and (38), $\dot{V}$ becomes

(39)V˙≤∑k=1Lr˜k⊤(t)ζk(t)+∑q=1LPkqQr˜k(t)−ψk(t,r˜k(t))=∑k=1L∑q=1LPkqr˜k⊤(t)Qr˜k(t)+∑k=1Lr˜k⊤(t)ζk(t)(40)−c∑k=1L∑j=1nr˜kj(t)(er˜kj(t)−1)er˜kj(t)(τf−t)=r˜⊤(t)(P⊗Q)r˜(t)−∑k=1L∑j=1nr˜kj(t)(er˜kj(t)−1)er˜kj(t)(τf−t)(41)+r˜⊤(t)ζ(t)(42)≤−c∑k=1L∑j=1n|r˜kj(t)|(e|r˜kj(t)|−1)e|r˜kj(t)|(τf−t)+r˜⊤(t)ζ(t)

with $\zeta \left(t\right)={\left[\zeta_{1}^{⊤}\left(t\right),\zeta_{2}^{⊤}\left(t\right),\cdots ,\zeta_{L}^{⊤}\left(t\right)\right]}^{⊤}$ and ${\tilde{r}}^{⊤}\left(t\right)={\left[{\tilde{r}}_{1}^{⊤}\left(t\right),{\tilde{r}}_{2}^{⊤}\left(t\right),\cdots ,{\tilde{r}}_{L}^{⊤}\left(t\right)\right]}^{⊤}$.

Similarly as in Theorem 1, we can write

(43) $$\begin{array}{c} \dot{V}\leq -c\sqrt{\frac{2V}{n}}\frac{\left(e^{\sqrt{\frac{2V}{n}}}-1\right)}{e^{\sqrt{\frac{2V}{n}}}\left(\tau_{f}-t\right)}+{\tilde{r}}^{⊤}\left(t\right)\zeta \left(t\right) \end{array}$$

Similarly, with the coupling control (32), for $t>\tau_{f}$ we can see that: $\dot{V}\leq {\tilde{r}}^{⊤}\left(t\right)\zeta \left(t\right)$.

Therefore, the MAS (8) becomes PTP using control (32) with output $\tilde{r}\left(t\right)$ and external input ζ(t). If ζ(t)=0, and using Corollary 1, the MAS (8) undergoes synchronization in a predefined time, which completes the proof. □

Now, in order to get rid of the Lipschitzness condition of the function ϕ(·), the following result is developed for MAS (8) to be PTP, along with agents’ synchronization in a predefined time using the developed adaptive state-feedback control.

**Theorem** **4.** *Consider the MAS (8) with the following adaptive state-feedback coupling control*

(44) $$\begin{array}{cc} \mu_{k}= & \left\{\begin{array}{c} \zeta_{k}+\sum_{q=1}^{L}P_{kq}\left(t\right)Q{\tilde{r}}_{k}-\psi_{k}\left(t,{\tilde{r}}_{k}\right),\;\;\text{if}\;\;t\in \left[t_{0},\tau_{f}\right)\\ \zeta_{k}+\sum_{q=1}^{L}P_{kq}\left(t\right)Q{\tilde{r}}_{k},\;\;\;\;\;\;\;\;\;\;\;\;\;\;\;\;\;\;\;\text{otherwise} \end{array}\right. \end{array}$$

*and*

(45) $$\begin{array}{c} {\dot{P}}_{kq}\left(t\right)=m_{kq}{\left({\tilde{r}}_{k}-{\tilde{r}}_{q}\right)}^{⊤}Q\left({\tilde{r}}_{k}-{\tilde{r}}_{q}\right)+2m_{kq} \end{array}$$

*where $m_{kq}=m_{qk}>0\in R$, $Q\in R^{n\times n}>0$, and $P_{kq}\left(t\right)\in R^{L\times L}$ is a time-varying Laplacian of graph O, defined such that: $P_{kq}\left(t\right)=P_{qk}\left(t\right)\in R>0$ when an edge exists between agent k and q (q≠k), otherwise $P_{kq}\left(t\right)=P_{qk}\left(t\right)=0$(q≠k), and*

(46) $$P_{kk}\left(t\right)=-\sum_{q=1\;q\neq k}^{L}P_{kq}\left(t\right).$$

*Then, the MAS (8) will be PTP and if ζ(t)=0, then it undergoes synchronization in predefined-time, where $\zeta \left(t\right)={\left[\zeta_{1}^{⊤}\left(t\right),\zeta_{2}^{⊤}\left(t\right),\cdots ,\zeta_{l}^{⊤}\left(t\right)\right]}^{⊤}$.*

**Proof.**  Consider $V=\frac{1}{2}\sum_{k=1}^{L}{\tilde{r}}_{k}^{⊤}\left(t\right){\tilde{r}}_{k}\left(t\right)$ and $V^{\prime }=\sum_{k=1}^{L}\sum_{q\in N_{k}}^{}\frac{{\left(P_{kq}\left(t\right)-\alpha_{kq}\right)}^{2}}{4m_{kq}}$. Let us consider a positive storage function

(47)V1=V+V′(48)V1=12∑k=1Lr˜k⊤(t)r˜k(t)+∑k=1L∑q∈Nk(Pkq(t)−αkq)24mkq

where $\alpha_{kq}=\alpha_{qk}\geq 0\in R\;\left(q\neq k\right)$ is a constant to be selected later with $\alpha_{kq}=0$ if $P_{kq}\left(t\right)=0$, and $\alpha_{kk}=-\sum_{q=1\;q\neq k}^{L}\alpha_{kq}$, and $N_{k}$ is the set of agents connected to the agent *k*. Thus, the derivative of the storage function along the system trajectories (31) for $t\in [t_{0},\tau_{f})$ is given by

(49)V1˙=∑k=1Lr˜k⊤(t)r˜˙k(t)+∑k=1L∑q∈Nk(Pkq(t)−αkq)2mkqP˙kq(t)=∑k=1L∑q=1LPkq(t)r˜k⊤(t)Qr˜k(t)+∑k=1Lr˜k⊤(t)ζk(t)−c∑k=1L∑j=1nr˜kj(t)(er˜kj(t)−1)er˜kj(t)(τf−t)+l∑k=1Lr˜k⊤(t)r˜k(t)+12∑k=1L∑q∈Nk(Pkq(t)−αkq)(r˜k−r˜q)⊤Q(r˜k−r˜q)(50)+∑k=1L∑q∈Nk(Pkq(t)−αkq)

Since

(51) $$\begin{array}{c} \sum_{k=1}^{L}\sum_{q\in N_{k}}^{}\left(P_{kq}\left(t\right)-\alpha_{kq}\right){\left({\tilde{r}}_{k}-{\tilde{r}}_{q}\right)}^{⊤}Q\left({\tilde{r}}_{k}-{\tilde{r}}_{q}\right)\\ =-2\sum_{k=1}^{L}\sum_{q=1}^{L}\left(P_{kq}\left(t\right)-\alpha_{kq}\right){\tilde{r}}_{k}^{⊤}\left(t\right)Q{\tilde{r}}_{q}\left(t\right) \end{array}$$

Now $\dot{V}$ dynamics becomes

V1˙≤∑k=1L∑q=1Lαkqr˜k⊤Qr˜q−c∑k=1L∑j=1n|r˜kj|(e|r˜kj|−1)e|r˜kj|(τf−t)(52)+l∑k=1Lr˜k⊤r˜k+∑k=1Lr˜k⊤ζk+∑k=1L∑q∈Nk(Pkq(t)−αkq)≤r˜⊤(α⊗Q+lIL×n)r˜+r˜⊤ζ−c2Vn(e2Vn−1)e2Vn(τf−t)(53)+∑k=1L∑q∈Nk(Pkq(t)−αkq)

where $\alpha =\left[\alpha_{kq}\right]\in R^{L\times L}$ and $\zeta ={\left[\zeta_{1}^{⊤},\zeta_{2}^{⊤},\cdots ,\zeta_{L}^{⊤}\right]}^{⊤}$. The further part of the proof is as same as done in the paper [51]. Let us recall it. Let there exist a unitary matrix $U=\left(u_{1},u_{2},\cdots ,u_{L}\right)\in R^{L\times L}$ such that $U^{⊤}\alpha U=P=\mathrm{diag}\left(p_{1},p_{2},\cdots ,p_{L}\right)\in R^{L\times L}$ where $0=p_{1}>p_{2}\geq p_{3}\geq \cdots \geq p_{L}$. Let $D\left(t\right)={\left[d_{1}^{⊤}\left(t\right),d_{2}^{⊤}\left(t\right),\cdots ,d_{L}^{⊤}\left(t\right)\right]}^{⊤}=\left(U^{⊤}⊗I_{n}\right)\tilde{r}\left(t\right).$ Since, $u_{1}=\frac{1}{\sqrt{L}}{\left[1,1,\cdots ,1\right]}^{⊤}$, one can say that $d_{1}\left(t\right)=\left(u_{1}^{⊤}⊗I_{n}\right)\tilde{r}\left(t\right)=0$. Then,

r˜⊤(t)(α⊗Q+lIL×n)r˜(t)(54) =r˜⊤(t)[(U⊗In)(P⊗Q)(U⊤⊗In)]r˜(t)+ηr˜⊤(t)r˜(t)(55) =D⊤(t)(P⊗Q)D(t)+lr˜⊤(t)r˜(t)(56) ≤p2D⊤(t)(Il⊗Q)D(t)+lr˜⊤(t)r˜(t)(57) =r˜⊤(t)(p2Il⊗Q+lIl×n)r˜(t).

We choose $\alpha_{kq}$ sufficiently large such that $k_{2}\lambda_{s}\left(Q\right)+l\leq 0$ and a time $0<t_{0}\in R$ that satisfies $P_{kq}\left(t\right)\geq \alpha_{kq}$ for all (k,q)∈D and $t\geq t_{0}$. Hence, $\dot{V}$ dynamics becomes for all $t_{0}\leq t<\tau_{f}$

V˙≤r˜⊤(t)[(P(t)−α)⊗Q+α⊗Q+lIL×n]r˜(t)(58)−c2Vn(e2Vn−1)e2Vn(τf−t)+r˜⊤ζ(59)≤−c2Vn(e2Vn−1)e2Vn(τf−t)+r˜⊤ζ

 □

Applying adaptive coupling control (44), one can observe that the dynamics $\dot{V}$ becomes: $\dot{V}\leq {\tilde{r}}^{⊤}\left(t\right)\zeta \left(t\right)$ for $t>\tau_{f}$. Hence, with control (44) the MAS (8), under external input ζ(t) and output $\tilde{r}\left(t\right)$, is passive. Additionally, the MAS (8) undergoes predefined-time synchronization if the external input ζ(t)=0. The proposed theoretical results are validated through the following examples.

**Example** **1.** *Consider the system of Chua’s circuit (1). The function ϕ(·) satisfies the Lipschitz condition*

(60) $$∥\phi \left(x_{1}\right)-\phi \left(x_{2}\right)∥\leq l∥x_{1}-x_{2}∥$$

**Figure 1:**
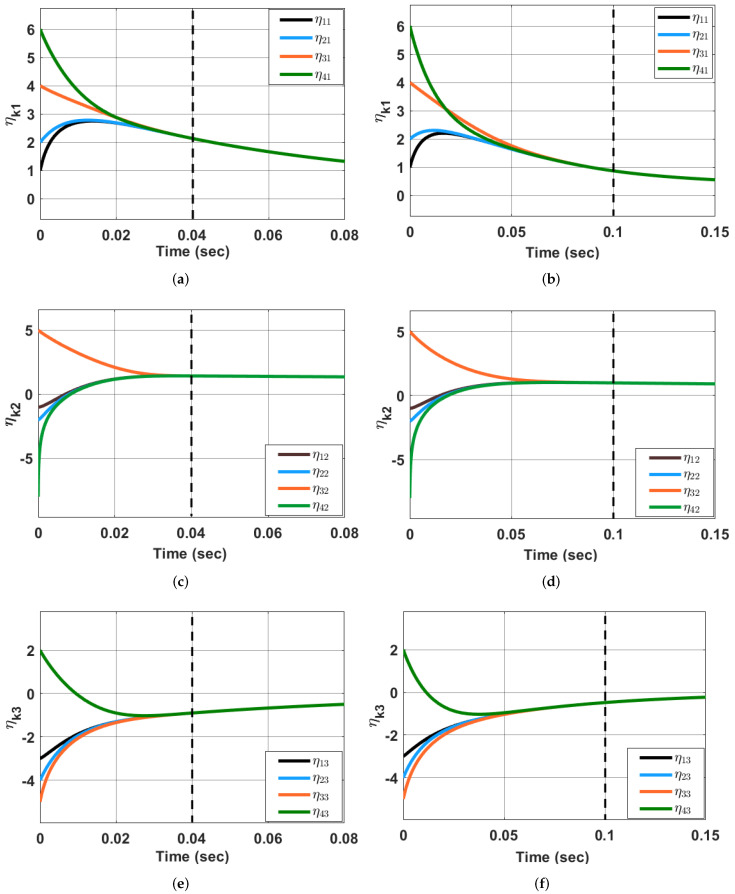
Synchronization of the states of MAS (1) using the control (32) (with ζ(t)=0) in predefined-time $\tau_{f}=0.04$ s (**a**,**c**,**e**) and $\tau_{f}=0.1$ s (**b**,**d**,**f**).

*for $x_{1},x_{2}\in R^{3}$, where l=29. Thus, from Theorem 3, the MAS (1) (with ζ(t)=0) undergoes predefined-time synchronization using state-feedback coupling controller (32). Figure 1 shows the simulation results using Q=diag(4.5,6.7,8.9),S=40,c=2.8 and*

$$\begin{array}{c} P=\left\{\begin{array}{cccc} -0.8 & 0.4 & 0.4 & 0\\ 0.2 & -0.6 & 0.2 & 0.2\\ 0.2 & 0.2 & -0.5 & 0.1\\ 0.2 & 0.2 & 0.1 & -0.5 \end{array}\right\} \end{array}$$

*From Figure 1, one can visualize that the states of the agents converge with respect to each other in* $\tau_{f}=0.04$ *sec and* $\tau_{f}=0.1$ *sec, which is predefined. Further, the MAS (1) undergoes synchronization using the adaptive state-feedback controller (44) (when* ζ(t)=0*) in predefined-time considering* Q=diag(7.5,4.2,6.9),c=2.8, $m_{kq}=1$ and

$$\begin{array}{c} P\left(0\right)=\left[\begin{array}{cccc} -0.05 & 0.05 & 0 & 0\\ 0.05 & -0.03 & -0.02 & 0\\ 0 & -0.02 & -0.06 & 0.08\\ 0 & 0 & 0.08 & -0.08 \end{array}\right] \end{array}$$

**Figure 2:**
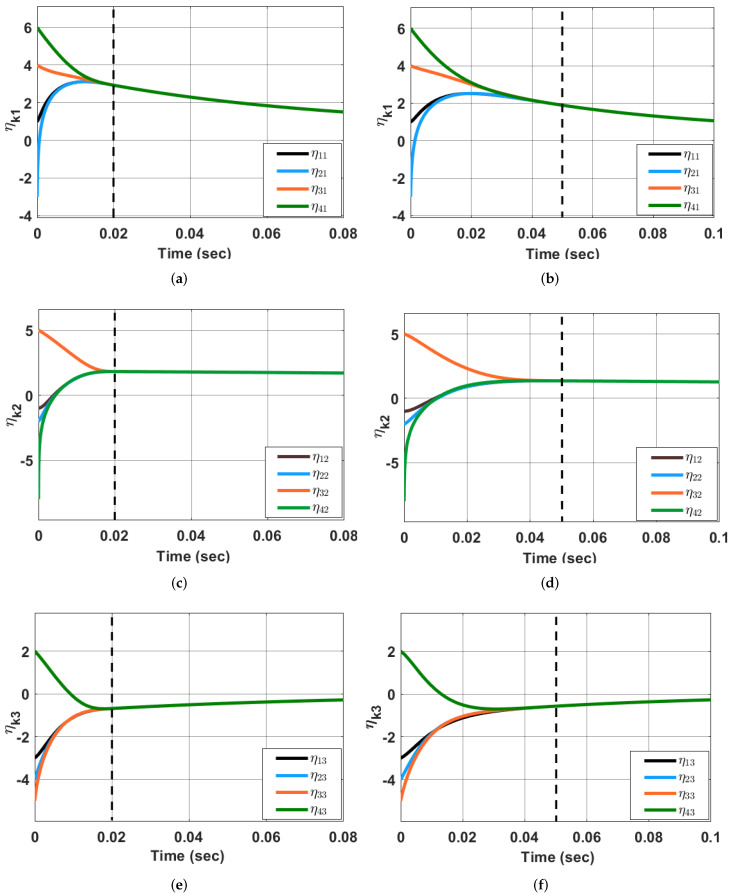
Synchronization of the states of MAS (1) using the adaptive control (32) and (44) (with ζ(t)=0) in predefined-time $\tau_{f}=0.02$ s (**a**,**c**,**e**) and $\tau_{f}=0.05$ s (**b**,**d**,**f**).

*The simulation results are shown in Figure 2.*

**Figure 3:**
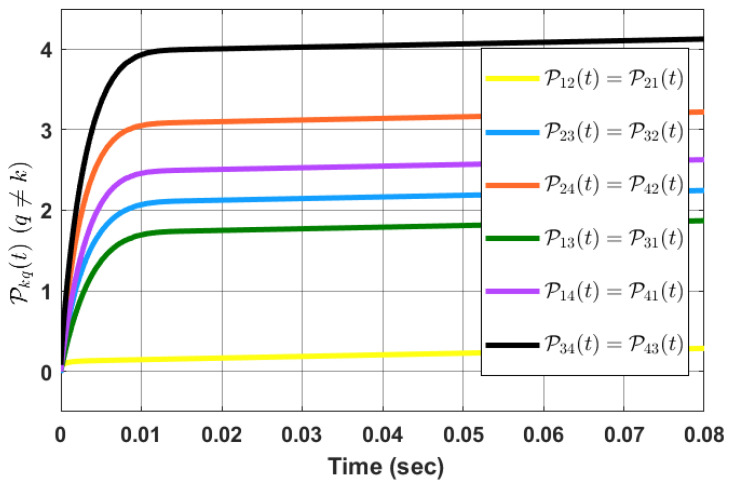
Evolution of $P_{kq}\left(t\right)$ with time.

*Simulation outcomes validate that agents’ synchronization with respect to each other occurs in the predefined time $\tau_{f}$. Figure 3 shows the evolution of $P_{kq}$ with time.*

**Example** **2.** *Consider the Kuramoto model (2). From Theorem 4, the MAS (2) undergoes synchronization using the adaptive state-feedback control (44) (when ζ(t)=0) in predefined-time considering ω=0.4, A=0.5, Q=1, c=2, $m_{pq}=3$ and*

$$\begin{array}{c} P\left(0\right)=\left[\begin{array}{cccccc} -5 & 1 & 1 & 1 & 1 & 1\\ 1 & -5 & 1 & 1 & 1 & 1\\ 1 & 1 & -5 & 1 & 1 & 1\\ 1 & 1 & 1 & -5 & 1 & 1\\ 1 & 1 & 1 & 1 & -5 & 1\\ 1 & 1 & 1 & 1 & 1 & -5 \end{array}\right] \end{array}$$

**Figure 4:**
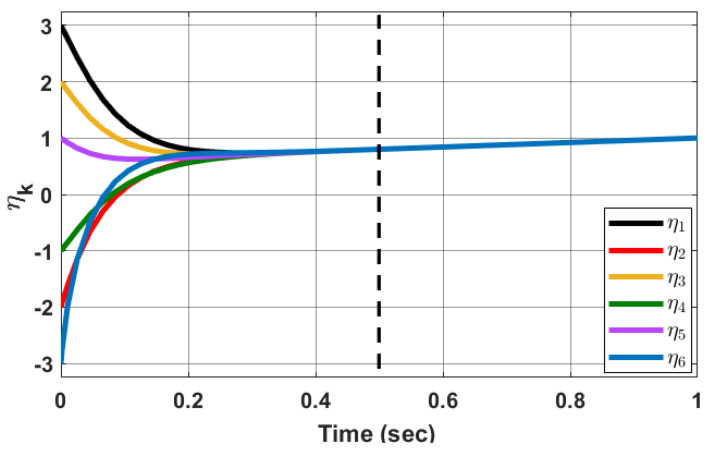
Synchronization of system (2) states with the adaptive state-feedback control (44) (ζ(t)=0) in time $\tau_{f}=0.5$ s.

**Figure 5:**
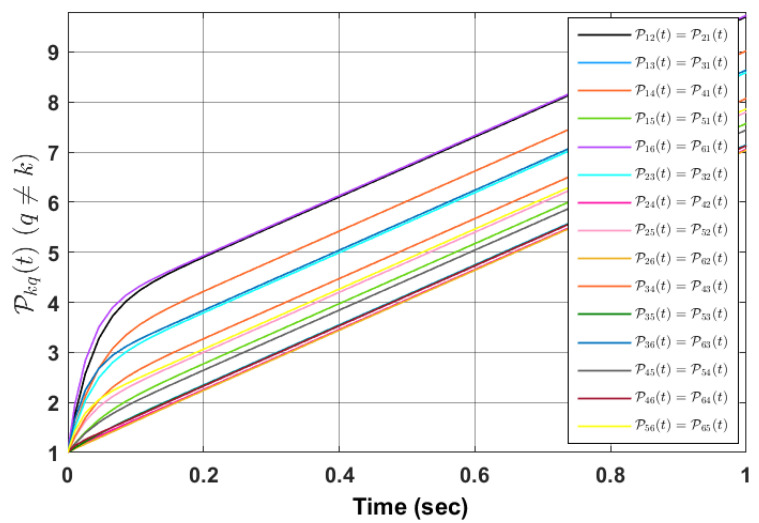
Evolution of $P_{kq}\left(t\right)$ with time.

*The simulation results are shown in Figure 4 for $\tau_{f}=0.5$ sec as the predefined time. Simulation results confirm that the state of the oscillators synchronizes with respect to each other in the predefined time, i.e., $\tau_{f}$. Figure 5 shows the evolution of $P_{kq}\left(t\right)$ with time.*

**Figure 6:**
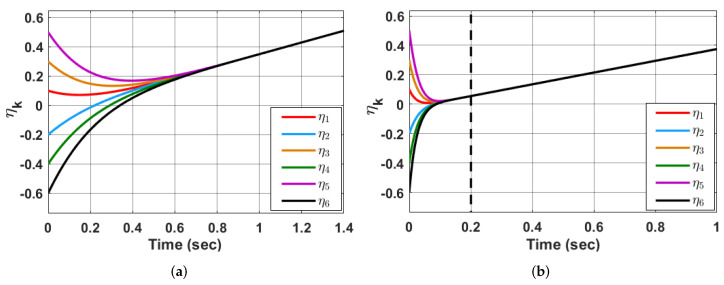

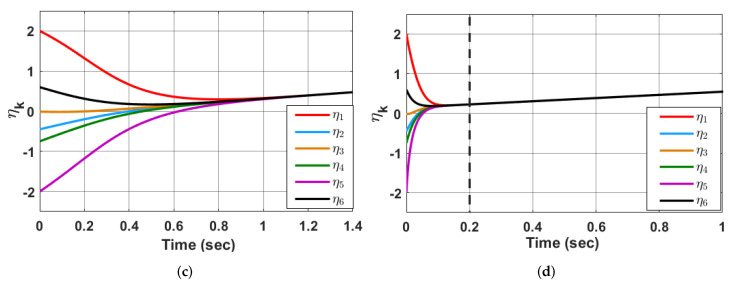
State evolutions of system (2) with time using the finite-time state-feedback controller designed in [51] with variations in the initial conditions (Finite-time synchronization) (**a**,**c**) and predefined-time state-feedback controller (32) with $\tau_{f}=0.2$ s with variation in initial conditions (**b**,**d**).

*Furthermore, a comparison is made with the finite-time state-feedback controller designed in [51] considering this Kuramoto model. Simulation results in Figure 6a,c shows the finite-time synchronization of the agents where the time of convergence (convergence time) changes with the change in the initial conditions. As in Figure 6a, the initial conditions of the 6 agents of Kuramoto model (2) are [0.1,−0.2,0.3,−0.4,0.5,−0.6] respectively, and the time of convergence using the state feedback controller in [51] is around 0.9 s. Whereas in Figure 6c, as the initial conditions of the agents are changed to [2,−0.45,−0.01,−0.75,−2,0.6], convergence time changes to 1.2 s. While in Figure 6b,d, using the controller (32), with the predefined-time chosen as 0.2 s, synchronization occurs at 0.2 s (predefined-time chosen a priori), irrespective of the change in initial conditions. The initial conditions chosen for simulation in Figure 6a,b are [0.1,−0.2,0.3,−0.4,0.5,−0.6] and for Figure 6c,d are [2,−0.45,−0.01,−0.75,−2,0.6] respectively for agents 1 to 6 of Kuramoto model (2). Thus, one can say that the proposed technique provides better results than finite-time techniques existing in the literature in the sense that the convergence time in the former case can be chosen in advance, while the convergence time in the latter case changes with the change in the initial conditions and cannot be chosen a priori.*

## 4. Conclusions

In the present paper, we have developed a predefined-time passivity notion, based on predefined-time stability, which is exploited for synchronization of nonlinear MAS. We have studied the tracking problem for a single agent and it is shown that tracking error dynamics is predefined-time passive using the designed control law (i) state feedback and (ii) adaptive state feedback, and later we have shown that tracking error goes to zero in the predefined time (which is chosen in advance), in the absence of external input. Further, we have extended it for nonlinear MAS where we have designed state feedback and adaptive state feedback protocols for synchronization of agents in the predefined time. A few examples were illustrated to show the validity of the theoretical results and a comparison with the finite-time passivity-based control scheme for MAS synchronization is shown.

As part of future work, it is proposed to do robustness analysis by considering uncertainty in the consensus of leader-follower-based problems. The proposed control scheme focuses on a completely connected graph, whereas it can be explored for directed graphs, switching graphs, etc. The paper mainly focuses on theoretical analysis and simulation results. Future work could explore the practical implementation of the proposed method in real-world systems and conduct experiments to validate its effectiveness. Also, developed notions can be explored to solve more realistic consensus problems with delay.

The proposed method assumes that all agents have the same dynamics and use the same control law, future work could investigate the extension of the proposed method to more general settings, such as agents with different dynamics or agents with different control laws. The paper compares the proposed method with existing synchronization methods based on their convergence rate and performance. However, it would be interesting to explore the proposed method for analyzing communication overhead, and fault tolerance related problems. 

## Data Availability

Not applicable.
